# External validation and updating of NTCP models for radiation pneumonitis: QUANTEC, Appelt, and a local simplified model

**DOI:** 10.3389/fonc.2026.1777999

**Published:** 2026-04-28

**Authors:** Zhi Chen, Heng Li, Lan Liang, Ming Fan, Shunping Huang, Peng Xue, Rui Kong, Zhenzhou Yang, Zhengjun Guo

**Affiliations:** 1 Department of Cancer Center, The Second Affiliated Hospital of Chongqing Medical University, Chongqing, China; 2 Department of Oncology and Hematology, West China Longquan Hospital, Sichuan University, The First People’s Hospital of Longquanyi District, Chengdu, China; 3 Department of Radiation Oncology, Precision Radiation in Oncology Key Laboratory of Sichuan Province, Sichuan Clinical Research Center for Cancer, Sichuan Cancer Hospital & Institute, Sichuan Cancer Center, University of Electronic Science and Technology of China, Chengdu, China; 4 Low-Altitude Science and Engineering College, Shandong University, Weihai, China; 5 Department of Oncology, the Third Affiliated Hospital of Chongqing Medical University, Chongqing, China

**Keywords:** DVH, external validation, lung cancer, mean lung dose, NLR, NTCP, radiation pneumonitis, SII

## Abstract

**Objective:**

To externally evaluate and update the QUANTEC and Appelt NTCP models for radiation pneumonitis (RP) in lung cancer patients treated with contemporary IMRT and multimodal therapy, and to preliminarily validate a simplified local model in an independent cohort.

**Methods:**

We retrospectively analyzed 580 lung cancer patients treated with thoracic IMRT between 2018 and 2023 as the development cohort. The QUANTEC and Appelt models were evaluated and locally updated using a closed testing procedure to determine the least extensive revision required. Clinical and DVH variables were standardized, and smoking status and pulmonary comorbidity were recoded according to published definitions. A final simplified local model (Model D) was developed using BIC-guided multivariable logistic regression with regularization. Performance was assessed by AUC, Brier score, calibration-in-the-large (CITL), calibration slope, Hosmer–Lemeshow test, and decision curve analysis. External validation of Model D was performed in 100 patients from an independent center using fixed coefficients.

**Results:**

Both the QUANTEC and Appelt models showed substantial calibration bias in the local cohort, with systematic underestimation of RP risk. Updating improved calibration as expected, with little change in discrimination. Model D, incorporating age, stage, smoking status, tumor location, pulmonary comorbidity, NLR, SII, V30, and MLD, showed the best apparent overall performance in the development cohort (AUC 0.708, Brier 0.215, CITL = 0, slope = 1, Hosmer–Lemeshow P = 0.599). In the external cohort, discrimination and prediction error were similar (AUC 0.718, 95% CI 0.576–0.831; Brier 0.207), although absolute RP risk was overestimated (CITL = −1.043, slope = 1.133, Hosmer–Lemeshow P < 0.001).

**Conclusions:**

The original QUANTEC and Appelt models underestimated RP risk in this contemporary IMRT cohort. Updating improved calibration, whereas discrimination changed little. Model D showed better apparent overall performance and preserved ranking ability in an independent external cohort. Calibration drift across centers suggests that simple recalibration may improve absolute risk estimation in new settings.

**Clinical trial registration:**

https://www.chictr.org.cn/hvshowproject.html?id=276191&v=1.1, identifier ChiCTR2500102055.

## 1 Introduction

Radiation pneumonitis (RP) is a frequent and clinically significant complication of thoracic radiotherapy, restricting dose escalation and adversely affecting both treatment efficacy and quality of life in lung cancer patients (1). RP risk is determined not only by pulmonary dose distribution but also by patient- and treatment-related factors such as age, smoking history, chemotherapy, and immunotherapy (2–4). To enable pretreatment risk assessment and optimize radiotherapy planning, normal tissue complication probability (NTCP) models have been proposed that integrate dosimetric and clinical variables (5).

The QUANTEC (Quantitative Analysis of Normal Tissue Effects in the Clinic) project established a seminal model using mean lung dose (MLD) as the sole predictor, recommending MLD <20 Gy to limit symptomatic RP (grade ≥2) risk to approximately 20% (6, 7). However, this dose-only model overlooks individual heterogeneity. Appelt et al. subsequently incorporated smoking, pulmonary comorbidities, age, and chemotherapy sequence to create a multifactorial model that improved personalized prediction (8). Yet, both models were derived from three-dimensional conformal radiotherapy cohorts, necessitating reassessment in the era of modern intensity-modulated radiotherapy (IMRT) and combined therapies (8).

With the widespread adoption of IMRT, concurrent chemoradiotherapy, and immunotherapy, historical NTCP models derived from earlier three-dimensional conformal radiotherapy cohorts may no longer provide accurate absolute RP risk estimates in contemporary practice. Changes in dose distribution, case mix, systemic treatment exposure, and supportive care are likely to have altered not only baseline RP incidence but also the effects of individual predictors. As a result, coefficients derived from older treatment-era populations may not be directly transportable to modern IMRT-based cohorts. Although previous external studies have shown that recalibration or variable reduction can improve model performance, substantial inter-population and treatment heterogeneity continues to limit the generalizability of existing models and supports the need for local reassessment and adaptation (9).

In this study, we retrospectively analyzed clinical and dosimetric data from lung cancer patients treated with IMRT and concurrent chemoradiotherapy, with or without immunotherapy, at our center between 2018 and 2023. We first externally evaluated the QUANTEC and Appelt NTCP models using a closed testing framework to assess discrimination, calibration, and overall fit, and to determine the least extensive level of updating required, ranging from simple recalibration to more substantial revision only when justified by model misfit. Because statistical updating alone may not fully address shifts in predictor effects under contemporary multimodal treatment, we further simplified the Appelt framework to derive a more parsimonious and clinically applicable RP prediction model integrating mean lung dose with key clinical factors. Its performance was subsequently examined in an independent external cohort of 100 patients using fixed coefficients without re-estimation, providing a preliminary assessment of transportability rather than evidence of broad generalizability.

## 2 Materials and methods

### 2.1 Study design

This study followed the Transparent Reporting of a multivariable prediction model for Individual Prognosis Or Diagnosis plus Artificial Intelligence (TRIPOD+AI) statement and was classified as a type 3 study, including model updating and independent external validation (10).

### 2.2 Study cohorts

The primary study cohort was derived from an institutional “RT-lung” multimodal database of lung cancer radiotherapy. The study was prospectively registered on the Biomedical Artificial Intelligence Research Registry (ID: uEJKlr) (11). All patients in the development cohort underwent thoracic intensity-modulated radiotherapy (IMRT) between March 1, 2018, and July 1, 2023. An independent external validation cohort was collected from another tertiary cancer center during a separate study period. Clinical variables were extracted from the electronic medical record system, and dose–volume histogram (DVH) parameters were exported from the radiotherapy treatment planning system using the same variable definitions and extraction workflow as in the development cohort. To ensure methodological comparability, the same inclusion and exclusion criteria, endpoint definition for grade ≥2 radiation pneumonitis, and planned post-radiotherapy follow-up schedule were applied to the external cohort. Patients in the external cohort also received thoracic IMRT within the same dose-fractionation framework as the development cohort.

Inclusion criteria: (i) age ≥18 years; (ii) pathologically confirmed primary lung cancer (adenocarcinoma, squamous cell carcinoma, or small-cell lung cancer); (iii) availability of follow-up chest CT scans at 1, 3, and 6 months (± 15 days) after radiotherapy.

Exclusion criteria: (i) interruption of radiotherapy >1 week or incomplete treatment; (ii) absence of baseline chest CT within 1 month prior to radiotherapy or missing the required post-treatment chest CT follow-up at 1, 3, and 6 months (± 15 days);

(iii) postoperative patients receiving irradiation to lymphatic drainage regions only.

In total, 580 patients were included in the development cohort, and 100 patients were included in the external validation cohort. The study was reviewed by the institutional review board, with exemption from additional ethical approval. Collected variables included demographics, tumor characteristics, occurrence of radiation pneumonitis (RP), treatment-related parameters (DVH, chemotherapy/immunotherapy regimens), hematological inflammatory markers, and follow-up outcomes.

### 2.3 Treatment regimen

All patients were treated with IMRT at a total dose of 45–60 Gy, delivered in 25–30 fractions, corresponding to a physical dose-per-fraction range of 1.5–2.4 Gy. Radiotherapy plans were individualized by radiation oncologists based on tumor volume, clinical stage, and pulmonary function. Some patients also received concurrent, induction, or adjuvant chemotherapy and/or immunotherapy. Chemotherapy regimens mainly consisted of platinum-based doublets combined with paclitaxel, gemcitabine, or pemetrexed. Immunotherapy consisted primarily of PD-1/PD-L1 inhibitors administered during concurrent, consolidation, or adjuvant phases. All treatment decisions were made by a multidisciplinary team (MDT).

### 2.4 Diagnosis and follow-up of RP

All patients were followed according to a standardized schedule, with chest CT scans performed at 1, 3, and 6 months (± 15 days) after radiotherapy. Although routine clinical follow-up could continue beyond 6 months, the study endpoint was predefined as grade ≥2 radiation pneumonitis (RP) occurring within the first 6 months after radiotherapy, because the present analysis focused on early RP-related radiographic and clinical changes during this period. RP was graded according to the National Cancer Institute Common Terminology Criteria for Adverse Events (CTCAE), version 5.0 (12). To improve diagnostic consistency, two experienced radiologists independently reviewed CT images based on the radiographic grading standards for radiation-induced lung injury (RGS), and discrepancies were resolved by a third senior expert (**Supplementary Table 1**; **Supplementary Figure S1**) (13).

### 2.5 Model development and updating

In the development cohort, the original QUANTEC and APPELT models were first evaluated and locally updated when indicated, whereas the independent external cohort was reserved for external assessment of the final fitted simplified model only. We validated and updated the QUANTEC model and the Appelt framework, the latter including updated Appelt models (Models A–C). The need for updating was assessed using the closed testing procedure proposed by Vergouwe et al., including recalibration-in-the-large, recalibration, and full model revision. In this context, model revision refers to re-estimation of all predictor–outcome associations. The QUANTEC model was originally derived from pooled re-analysis of 10 retrospective cohorts using mean lung dose (MLD) as the sole predictor (6). For clarity and to avoid misinterpretation of coefficient direction, the original equations are presented below in standard logistic form, with the linear predictor (LP) defined separately:

QUANTEC model:


$$NTCP_{QUANTEC}={\left[1+\exp(-LP_{QUANTEC})\right]}^{-1}$$

$$LP_{QUANTEC}=-3.87+0.126\times MLD$$

The Appelt model further incorporated clinical variables including smoking status, age, pulmonary comorbidity, sequencing of chemotherapy, and tumor location, and is presented in the same form (8):

Appelt model:


$${\text{NTCP}}_{\text{APPELT}}={\left[1+\text{exp}(-{\text{LP}}_{\text{APPELT}})\right]}^{-1}$$

$${\text{LP}}_{\text{APPELT}}=-4.76+0.138\times \text{MLD}-0.48\times \text{current smoker}-0.37\times \text{former smoker}+0.82\times \text{pulm CoMorb}+0.51\times \text{old Age}+0.47\times \text{sequ Chemo}+0.63\times \text{MidOrInf}$$

Separate from the updating of these existing models, a final simplified local model (Model D) was developed to derive a more parsimonious and clinically applicable prediction model for the contemporary IMRT cohort. Model D was informed by the Appelt framework but was not obtained through direct reduction of the original Appelt model. Candidate predictors for Model D were prespecified from three sources: variables included in the original Appelt framework, clinically established RP-related dosimetric factors, and inflammatory biomarkers available in our cohort. Univariable logistic regression was used for exploratory assessment only and was not the sole basis for variable retention. Final predictor selection was performed within a multivariable logistic regression framework using Bayesian Information Criterion (BIC)-guided simplification with regularization, with preference given to parsimonious models that improved overall model fit while remaining clinically interpretable. Accordingly, Model D represents the final multivariable logistic regression model selected from the candidate model forms on the basis of overall fit, parsimony, and clinical interpretability, and was therefore carried forward for external validation. No missing data were present in the variables used for model development and external validation after cohort assembly. DVH parameters were directly exported from the radiotherapy treatment planning system, and only patients with complete clinical, dosimetric, and follow-up information required for the prespecified analyses were retained.

To explore robustness and parsimony, alternative model forms were compared during model development, including standard logistic regression, spline-expanded models, ridge regression, and lasso regression, in order to evaluate different levels of model complexity and shrinkage when deriving the final simplified local model. Candidate clinical and dosimetric variables were standardized before analysis where appropriate. Variable handling was as follows: age was treated as a continuous variable, and smoking status was categorized as never smoker, former smoker (≥3 months cessation), or current smoker during treatment. Potential non-linearity of age was explored using restricted cubic splines. As no clear evidence of non-linearity was observed, age was retained as a linear continuous predictor in the final model.

All DVH-derived dosimetric variables, including MLD and V30, were based on physical dose (Gy) directly exported from the radiotherapy treatment planning system; no EQD2 or other biologically equivalent dose conversion was applied. V30 was defined as the percentage volume of the total bilateral lung receiving ≥30 Gy. Alternative volumetric dose metrics were also examined during variable screening, and V30 was retained in the final model under the prespecified BIC-guided selection framework. Pulmonary comorbidity was coded as a binary variable (yes/no), including chronic obstructive pulmonary disease, pulmonary fibrosis, and other clinically documented chronic pulmonary diseases. Because MLD and V30 both reflect lung dose burden, potential collinearity between these variables was considered during model building. Both variables were retained only if their joint inclusion improved model fit under the BIC-guided framework, suggesting complementary rather than fully redundant predictive information. Under this procedure, NLR, SII, and V30 were retained because they provided additional predictive information beyond the original Appelt variables and MLD. Candidate predictors were entered into the multivariable modeling framework and sequentially reduced under the BIC criterion, with attention to clinical interpretability and model stability. The final retained variables were those included in the most parsimonious model that achieved the best overall fit under this procedure.

### 2.6 Model performance evaluation

Model goodness-of-fit was assessed using log-likelihood (LL), Akaike Information Criterion (AIC), and Bayesian Information Criterion (BIC). Discrimination was quantified by the area under the receiver operating characteristic curve (AUC), and overall prediction error was measured with the Brier score. In the development cohort, bootstrap resampling with replacement (1,000 resamples) was applied after final model specification to estimate optimism-corrected AUC and to provide bootstrap-based internal validation for the final fitted simplified model. Specifically, after model updating or simplified model development had been completed, the final selected predictor set was held fixed, models were re-fitted in bootstrap samples of the same size as the original development cohort, and performance was evaluated in the original development dataset. Clinical utility was evaluated using decision curve analysis (DCA). In this study, DCA was used to assess whether the models could support identification of patients at sufficiently high predicted risk of grade ≥2 RP to justify intensified risk-adapted management, such as stricter lung dose constraints, more cautious treatment planning, or closer post-treatment surveillance. The threshold probability represents the predicted RP risk above which such additional management strategies would be considered; low-to-intermediate thresholds were regarded as the most clinically relevant in this setting.

Calibration was assessed mainly using calibration-in-the-large (CITL), calibration slope, and calibration plots, which were used to visualize agreement between predicted and observed event probabilities. The Hosmer–Lemeshow test was retained as a supplementary descriptive measure but was not used as the primary basis for judging calibration performance. For consistency, Hosmer–Lemeshow statistics before and after model updating were computed using the same probability-based grouping strategy and the same implementation throughout, with decile-based quantile bins and duplicate cut-points dropped when necessary. In addition, a dose–response curve between mean lung dose (MLD) and the probability of grade ≥2 radiation pneumonitis was constructed to explore the dose–toxicity relationship.

### 2.7 External validation

The final simplified model (Model D) was externally validated in an independent cohort of 100 patients. Regression coefficients were fixed, and no re-estimation was performed on the external dataset. Validation metrics included LL, AIC, BIC, AUC, Brier score, CITL, calibration slope, Hosmer–Lemeshow χ², and Nagelkerke R².

### 2.8 Software and implementation

All statistical analyses and graphical visualizations were performed in Python, primarily using the scikit-learn and statsmodels packages, together with the open-source GitHub toolkit TCP-NTCP-dose_var_model(GitHub - mbolt01/TCP-NTCP-dose_var_model: Python code used to model the variation in TCP and NTCP with dose fraction specific dose variations).

## 3 Results

### 3.1 Baseline characteristics

**Figure 1:**
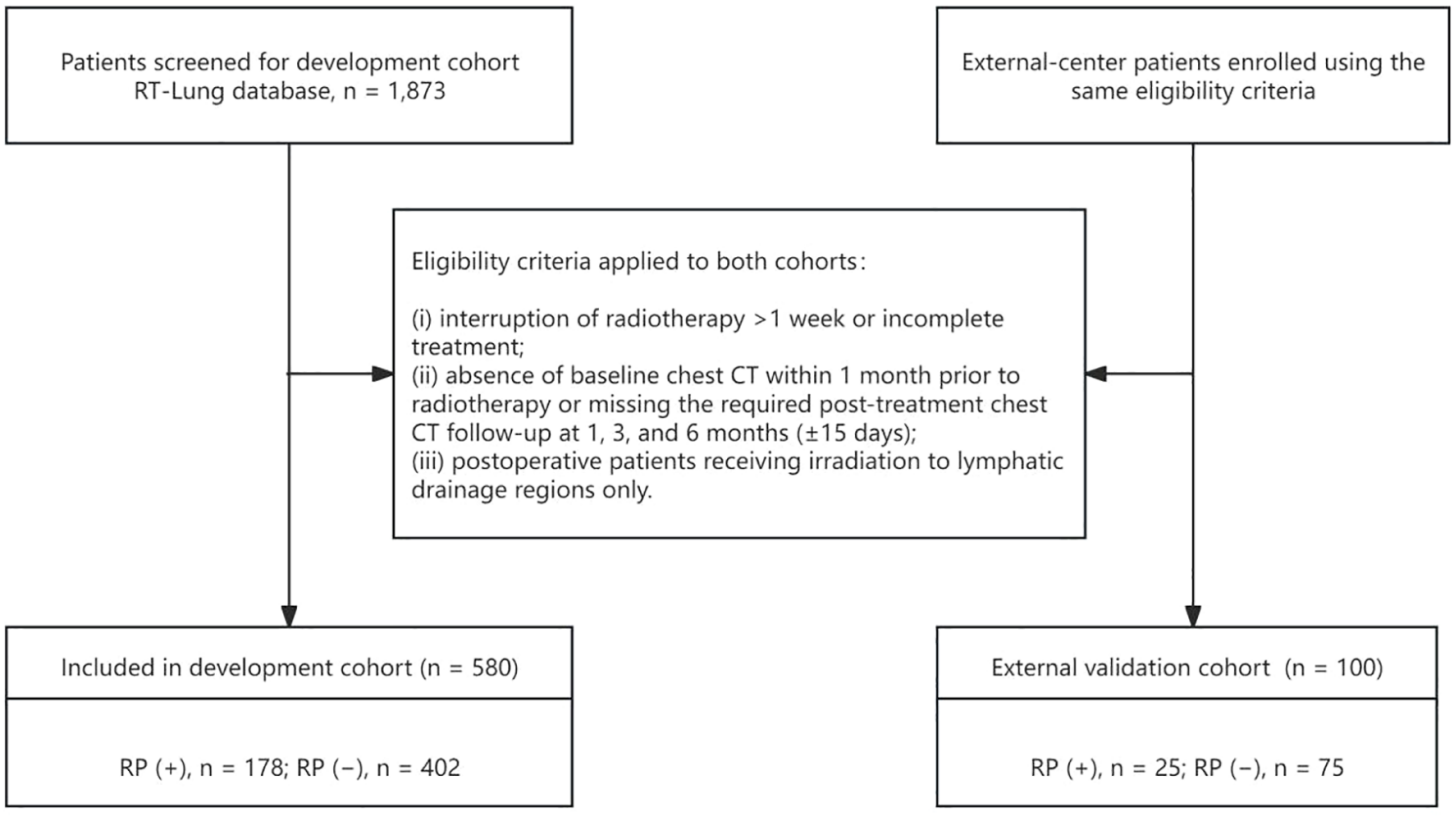
Flowchart of patient screening, application of eligibility criteria, and final inclusion in the development and external validation cohorts. RP, radiation pneumonitis; CT, computed tomography.

Patient screening and cohort assembly are illustrated in **Figure 1**. From the RT-Lung database, 1,873 patients were screened, of whom 1,263 had at least 6 months of follow-up. After exclusion of 683 ineligible patients, 580 patients were included in the development cohort. In addition, 100 eligible patients from an external center were included as the independent external validation cohort. Baseline characteristics are summarized in **Table 1**. The two cohorts were broadly comparable in sex, histology, and overall use of chemotherapy and immunotherapy, although a difference was observed in adjuvant chemotherapy.

**Table 1:** Baseline characteristics of patients in training and test cohorts.

Variables	Total (n=680)	Train (n=580)	Test (n=100)	p-value
Age	63.17 ± 9.56	63.23 ± 9.61	62.86 ± 9.28	0.718
SEX				1
1	540 (79.41)	461 (79.48)	79 (79.00)	
2	140 (20.59)	119 (20.52)	21 (21.00)	
Stage				<0.05
1	25 (3.68)	23 (3.97)	2 (2.00)	
2	29 (4.26)	20 (3.45)	9 (9.00)	
3	259 (38.09)	227 (39.14)	32 (32.00)	
4	367 (53.97)	310 (53.45)	57 (57.00)	
PS				0.465
0	204 (30.00)	170 (29.31)	34 (34.00)	
1	392 (57.65)	340 (58.62)	52 (52.00)	
2	84 (12.35)	70 (12.07)	14 (14.00)	
Smoking				0.906
current	9 (1.32)	8 (1.38)	1 (1.00)	
former	431 (63.38)	366 (63.10)	65 (65.00)	
never	240 (35.29)	206 (35.52)	34 (34.00)	
Diabete				0.42
0	599 (88.09)	508 (87.59)	91 (91.00)	
1	81 (11.91)	72 (12.41)	9 (9.00)	
Comorbidity				0.408
0	349 (51.32)	302 (52.07)	47 (47.00)	
1	331 (48.68)	278 (47.93)	53 (53.00)	
Histopathology				0.348
1	283 (41.62)	236 (40.69)	47 (47.00)	
2	217 (31.91)	191 (32.93)	26 (26.00)	
3	180 (26.47)	153 (26.38)	27 (27.00)	
Therapyside				0.096
1	285 (41.91)	235 (40.52)	50 (50.00)	
2	395 (58.09)	345 (59.48)	50 (50.00)	
MidOrInf				<0.05
1	338 (49.71)	276 (47.59)	62 (62.00)	
2	101 (14.85)	94 (16.21)	7 (7.00)	
3	241 (35.44)	210 (36.21)	31 (31.00)	
WBC	6.01 ± 2.95	6.06 ± 2.91	5.74 ± 3.15	0.349
N	4.17 ± 2.76	4.21 ± 2.70	3.91 ± 3.06	0.357
L	1.20 ± 0.49	1.21 ± 0.50	1.19 ± 0.45	0.723
NLR	4.24 ± 4.61	4.28 ± 4.61	4.01 ± 4.65	0.59
PLR	207.05 ± 172.55	207.26 ± 175.87	205.83 ± 152.68	0.933
LMR	3.28 ± 2.60	3.27 ± 2.65	3.35 ± 2.29	0.744
SII	971.03 ± 1835.42	956.14 ± 1604.07	1057.38 ± 2836.87	0.729
M	0.49 ± 0.30	0.49 ± 0.31	0.46 ± 0.25	0.329
PLT	209.55 ± 90.92	209.66 ± 90.81	208.88 ± 92.02	0.937
HB	120.20 ± 18.97	120.58 ± 18.88	118.03 ± 19.40	0.226
CRP	18.18 ± 31.52	18.31 ± 31.41	17.45 ± 32.27	0.806
LDH	186.93 ± 64.72	188.63 ± 65.87	177.08 ± 56.91	0.069
PTV	211.77 ± 421.80	214.84 ± 412.02	193.99 ± 476.49	0.681
Lungs	3085.14 ± 1411.41	3107.63 ± 1480.08	2954.69 ± 911.47	0.166
V5%	37.47 ± 12.53	38.07 ± 12.69	33.99 ± 11.02	<0.05
V20%	18.61 ± 10.67	18.93 ± 11.22	16.75 ± 6.41	<0.05
V30%	17.99 ± 12.60	15.97 ± 18.72	19.68 ± 19.19	0.477
MLD	9.89 ± 4.03	9.90 ± 4.14	9.87 ± 3.40	0.931
Chemotherapy				0.08
No	186 (27.35%)	151 (26.03%)	35 (35.00%)	
Yes	494 (72.65%)	429 (73.97%)	65 (65.00%)	
Immunotherapy				0.58
No	320 (47.06%)	276 (47.59%)	44 (44.00%)	
Yes	360 (52.94%)	304 (52.41%)	56 (56.00%)	

Values are presented as mean ± standard deviation (SD) or number (%).Abbreviations: PS, performance status; WBC, white blood cell count; N, neutrophil; L, lymphocyte; NLR, neutrophil-to-lymphocyte ratio; PLR, platelet-to-lymphocyte ratio; LMR, lymphocyte-to-monocyte ratio; SII, systemic immune-inflammation index; M, monocyte; PLT, platelet; HB, hemoglobin; CRP, C-reactive protein; LDH, lactate dehydrogenase; PTV, planning target volume; MLD, mean lung dose.

Overall, 680 patients were included, with a median age of 64 years (range, 24–86 years); 540 (79.4%) were male. Symptomatic radiation pneumonitis (RP, grade ≥2) occurred in 203 patients (29.9%), including 141 grade II (69.5%), 49 grade III (24.1%), 12 grade IV (5.9%), and 1 grade V event (0.5%; death on day 12 after radiotherapy). In the development cohort, 178 events occurred (123 grade II [69.1%], 43 grade III [24.2%], 11 grade IV [6.2%], and 1 grade V [0.6%]). In the external cohort, 25 events were observed (18 grade II [72.0%], 6 grade III [24.0%], and 1 grade IV [4.0%]), with no grade V event.

Regarding treatment, 494 of 680 patients (72.65%) received chemotherapy, including induction in 147 (21.62%), concurrent in 243 (35.74%), and adjuvant in 96 (14.12%). Immunotherapy was administered to 360 patients (52.94%), including induction in 110 (16.18%), concurrent in 50 (7.35%), and adjuvant in 308 (45.29%). These treatment-phase categories were not mutually exclusive, because some patients received chemotherapy and/or immunotherapy in more than one treatment phase. Only key treatment variables are presented here; detailed regimen distributions are provided in **Supplementary Table 2**.

After standardized coding and BIC-guided multivariable simplification, the final simplified local model (Model D) retained stage, smoking status (current/former/never), tumor location (mid/inferior), comorbidity, age, NLR, SII, V30, and MLD (**Table 2**).

**Table 2:** Comparison of APPELT model parameters and performance (original, recalibrated, updated, simplified, and final models).

Variables	Original APPELT (original)	Recalibrated model (model A)	Updated model (model B)	Simplified model (model C)	Variables	Final model (model D)
Model parameters					Model parameters	
Intercept	–4.76	–1.7575	–1.127	–1.7422	Intercept	–0.8714
MLD	0.138	0.138	0.085	0.1637	MLD	0.1199
Smoke_former	–0.37	–0.37	–0.2279	–	Smoke_former	–0.0572
Smoke_current	–0.48	–0.48	–0.2956	–	Smoke_current	–2.15
Comorbidity	0.82	0.82	0.5051	–	Comorbidity	–0.0626
Age	0.51	0.51	0.3141	–	Age	0.0405
SeqChemo	0.47	0.47	0.2895	–	Stage	–0.2493
Loc_midinf	0.63	0.63	0.388	–	NLR	–0.1669
–	–	–	–	–	SII	0.0006
–	–	–	–	–	V30	0.0498
Model performance					Model performance	
AUC	0.6275	0.6275	0.6275	0.6659	AUC	0.7075
Hosmer–Lemeshow χ²	2614.2484	18.7945	8.4222	3.9343	Hosmer–Lemeshow χ²	6.4276
P value	<0.001	0.016	0.3934	0.863	P value	0.5994
Calibration					Calibration	
Calibration intercept	1.8048	–0.0445	0	0	Calibration intercept	0
Calibration slope	0.6159	0.6159	1	1	Calibration slope	1

Additional abbreviations: SeqChemo, sequential chemotherapy; Loc_midinf, mid–inferior tumor location; V30, percentage of lung volume receiving ≥30 Gy.

Model performance definitions are as in **Table 3**. A dash (–) denotes that the variable was not included in the corresponding model.

### 3.2 External validation and recalibration of the QUANTEC model

#### 3.2.1 Original QUANTEC model

**Figure 2:**
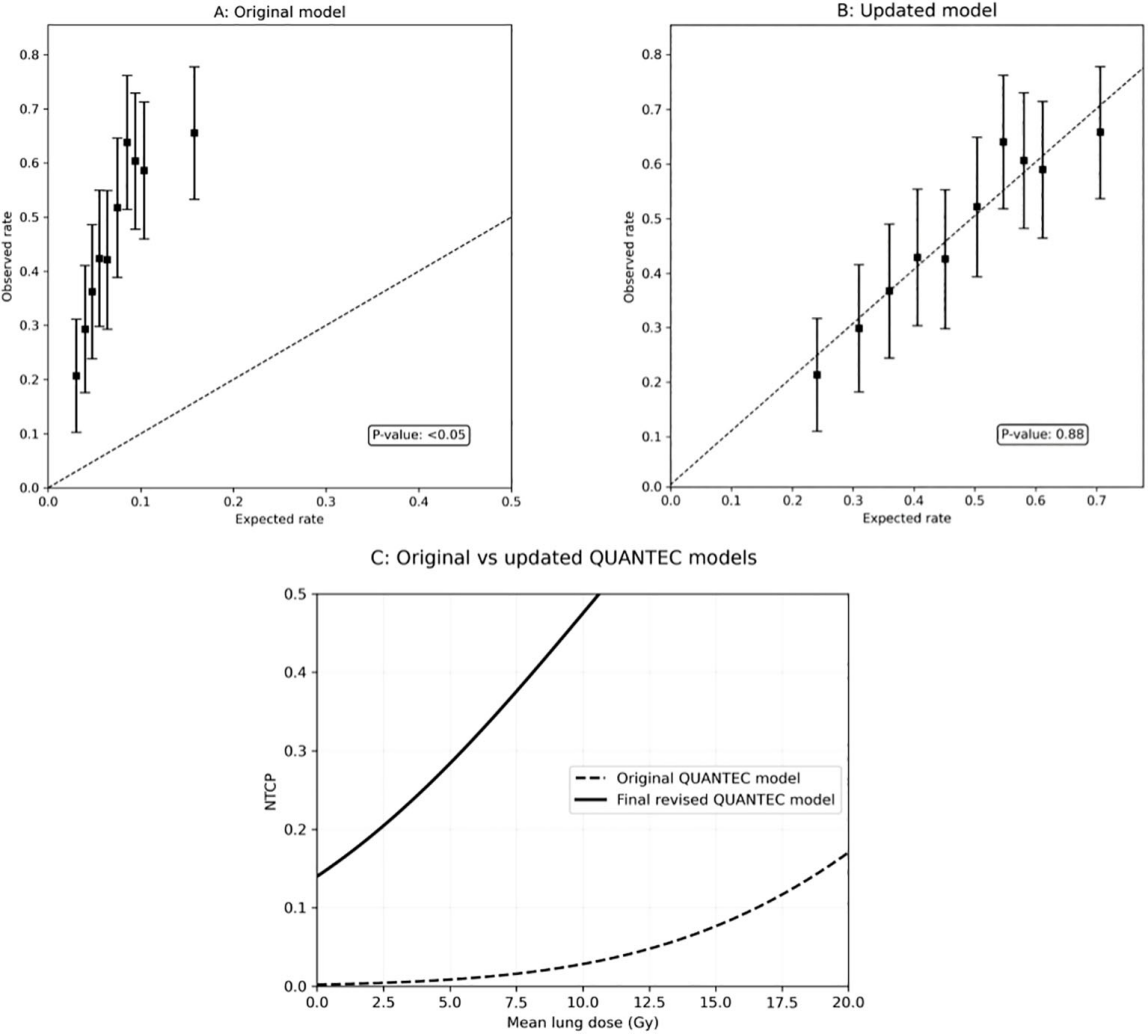
Calibration and dose–response relationship of the QUANTEC model. **(A)** Calibration curve of the original QUANTEC model, showing the agreement between predicted and observed rates of grade ≥2 radiation pneumonitis (Hosmer–Lemeshow test, P < 0.05). **(B)** Calibration curve of the updated QUANTEC model, showing improved agreement between predicted and observed rates (Hosmer–Lemeshow test, P = 0.88). **(C)** Dose–response relationship between mean lung dose (MLD) and the probability of grade ≥2 radiation pneumonitis for the original and updated QUANTEC models.

Closed testing indicated that the QUANTEC model required recalibration of both the intercept and the MLD coefficient (**Table 3**). In the development cohort, the original QUANTEC model showed modest discrimination but poor calibration, with an AUC of 0.666 (95% CI, 0.62–0.73), a Hosmer–Lemeshow χ² of 1379.619 (P<0.001), and clear underestimation of the observed RP risk (**Figure 2A**).

**Table 3:** Fitting and updating results of the QUANTEC model in the local cohort.

Variables	Original model	Recalibrated model	Final model
Model parameters			
Intercept	–3.87	–1.742	–1.652
MLD coefficient	0.126	0.164	0.155
Model performance			
AUC	0.666	0.666	0.666
Hosmer–Lemeshow χ²	1379.619	3.733	3.725
*p*-value	<0.001	0.88	0.881
Calibration			
Calibration intercept	3.287	0	0.007
Calibration slope	1.299	1	1.058

Model performance: AUC, area under the receiver operating characteristic curve; Hosmer–Lemeshow χ² and corresponding *P* values indicate the goodness-of-fit (higher *P* values suggest better calibration). Calibration intercept close to 0 and slope close to 1 indicate optimal calibration.

#### 3.2.2 Recalibrated QUANTEC model

After recalibration, discrimination remained unchanged (AUC = 0.666), whereas calibration improved substantially. The Hosmer–Lemeshow χ² decreased to 3.733 (P = 0.88), the calibration intercept changed from 3.287 to 0.007, and the calibration slope changed from 1.299 to 1.058 (**Figure 2B**; **Table 3**). These findings indicate that the major limitation of the original QUANTEC model in our cohort was systematic calibration error rather than poor patient ranking. As expected, recalibration improved agreement between predicted and observed risk without materially changing discrimination. The elevated pre-update Hosmer–Lemeshow χ² value was consistent with substantial baseline miscalibration of the original model in this cohort. Extended calibration statistics are provided in **Supplementary Table 4A**.

#### 3.2.3 Final updated QUANTEC model

Dose–response analysis further showed a positive association between MLD and the probability of grade ≥2 RP (**Figure 2C**). After coefficient updating and ridge-based shrinkage, the final updated QUANTEC model for the present cohort was derived as follows (**Table 3**):


$$NTCP_{QUANTEC}=\frac{1}{1+\text{exp}[-(-1.652+0.155\times MLD)]}$$

### 3.3 External validation, updating, and simplification of the APPELT model

#### 3.3.1 Original APPELT model

**Figure 3:**
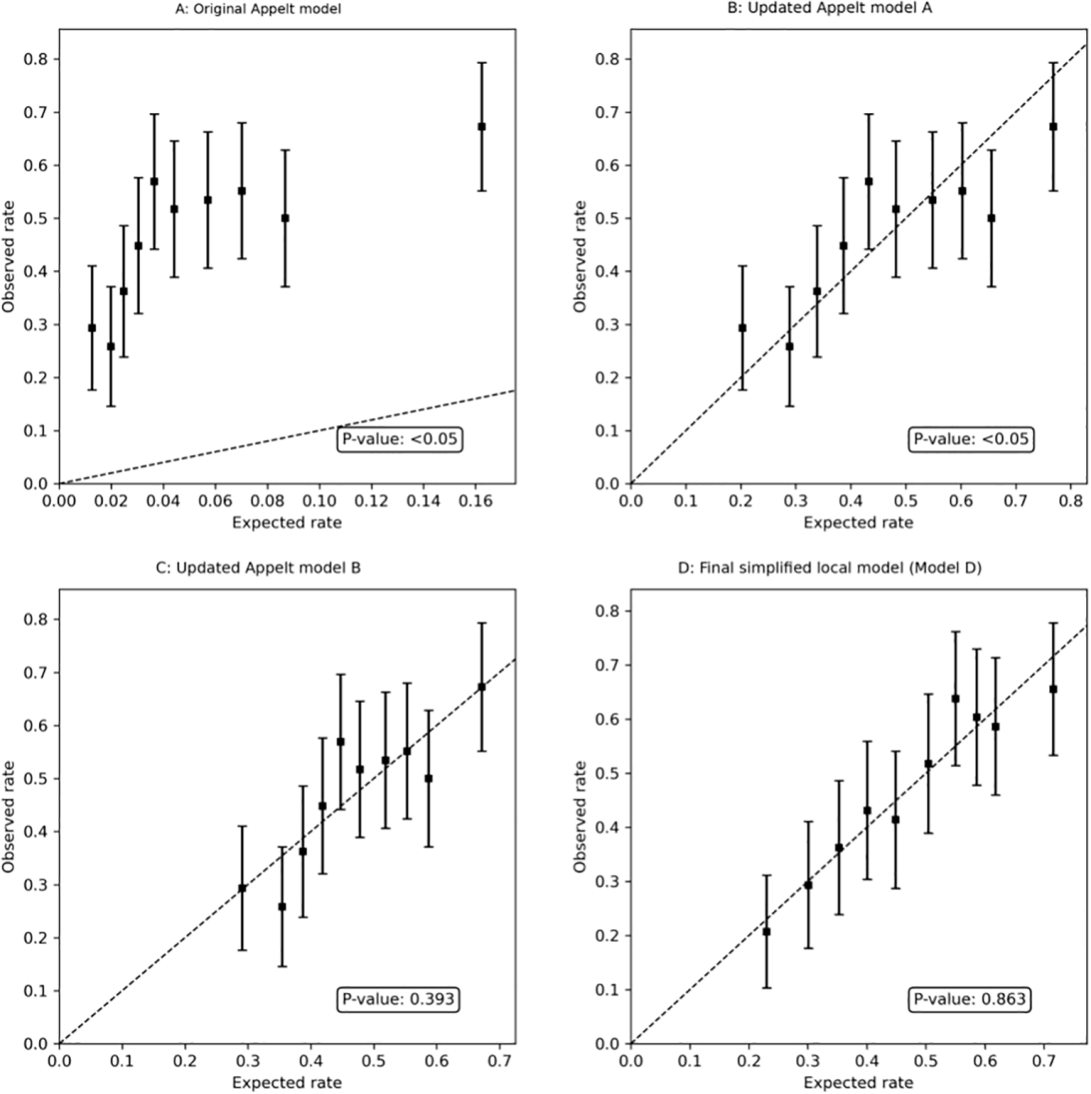
Calibration plots for sequential updating of the APPELT model in the development cohort. **(A)** Original Appelt model. **(B)** Updated Appelt model A after recalibration-in-the-large (intercept update only). **(C)** Updated Appelt model B after full recalibration (intercept and slope update). **(D)** Final simplified local model (Model D). The original model showed poor calibration with systematic underestimation of RP risk. Calibration improved progressively after model updating, with the final simplified local model showing the best overall agreement between predicted and observed risk.

In the development cohort, the original APPELT model showed limited discrimination and poor calibration. The AUC was 0.628, the Hosmer–Lemeshow χ² was 2614.25 (P<0.001), the calibration-in-the-large (CITL) was 1.805, and the calibration slope was 0.616, indicating substantial underestimation of RP risk and inadequate overall fit (**Table 2**; **Figure 3A**).

#### 3.3.2 Updated model A (intercept recalibration)

Following the closed testing procedure, recalibration-in-the-large was first performed by re-estimating the intercept only. Under this approach, discrimination remained unchanged, whereas baseline calibration improved. CITL improved from 1.805 to −0.045, and the Hosmer–Lemeshow χ² decreased to 18.79 (P = 0.016) (**Figure 3B**). However, slope underestimation persisted, indicating that correction of the baseline risk offset alone was insufficient.

#### 3.3.3 Updated model B (full recalibration)

Full recalibration was then performed by updating both the intercept and slope. This resulted in CITL = 0 and calibration slope = 1, with acceptable overall calibration (Hosmer–Lemeshow χ² = 8.42, P = 0.393) (**Figure 3C**). Discrimination again did not improve, which was expected because recalibration primarily affects absolute risk estimation and overall fit rather than patient ranking.

#### 3.3.4 Simplified model C

Based on BIC-guided simplification, the Appelt framework was then reduced to a simplified Appelt-derived model that retained only MLD. In this reduced model, the MLD coefficient increased from 0.138 to 0.164, the AUC increased to 0.666, and calibration was good (CITL = 0, slope = 1, Hosmer–Lemeshow χ² = 3.93, P = 0.863). Because both Model C and the updated QUANTEC model retained MLD as the sole predictor, their discrimination was expected to be similar. However, the two models arose from different updating pathways: the updated QUANTEC model was obtained by coefficient updating of the original single-predictor QUANTEC equation, whereas Model C resulted from BIC-guided simplification of the multivariable Appelt framework. Therefore, modest differences in coefficient estimates were methodologically plausible.

#### 3.3.5 Final simplified local model (model D)

To derive a locally adapted model for contemporary IMRT practice, candidate predictors were prespecified from the original Appelt framework together with clinically relevant inflammatory markers and conventional lung dose-volume parameters, and were then reduced using BIC-guided multivariable logistic regression. Within this process, inflammatory markers (NLR and SII) and dose-volume parameters (V30 and MLD) were retained together with key clinical variables, yielding the final simplified local model (Model D) (**Table 2**; **Figure 3D**).

In Model D, V30 was defined as the percentage volume of the total bilateral lung receiving ≥30 Gy, and pulmonary comorbidity was entered as a binary clinical variable. MLD and V30 were moderately to strongly correlated (Spearman ρ = 0.768, P < 0.001), but both variables were retained because their joint inclusion improved model fit while preserving parsimony, suggesting that they captured complementary aspects of lung dose burden, namely mean dose intensity and medium-dose volume exposure.

Model D included age, stage, smoking status, tumor location, pulmonary comorbidity, NLR, SII, V30, and MLD. In the development cohort, it achieved an AUC of 0.708, a Hosmer–Lemeshow χ² of 6.43 (P = 0.599), a CITL close to 0, and a calibration slope close to 1, indicating good discrimination and calibration. Detailed coefficients and extended performance metrics for all updating steps are shown in **Supplementary Table 4B**.

Compared with the final updated QUANTEC model, Model D showed better discrimination (AUC, 0.708 vs. 0.666) and greater explanatory power (Nagelkerke R², approximately 0.186 vs. 0.119). The final simplified local model for this cohort was expressed as follows:


$$NTCP_{Model\;D\;}={\left[1+exp(-(-0.8714+0.1199*MLD-0.0572*Smokeformer-2.1500*Smokcurrent-0.0626*Comorbidity+0.0405*oldage-0.2493*stage-0.1669*NLR+0.0006*SII+0.0498*V30))\right]}^{-1}$$

**Figure 4:**
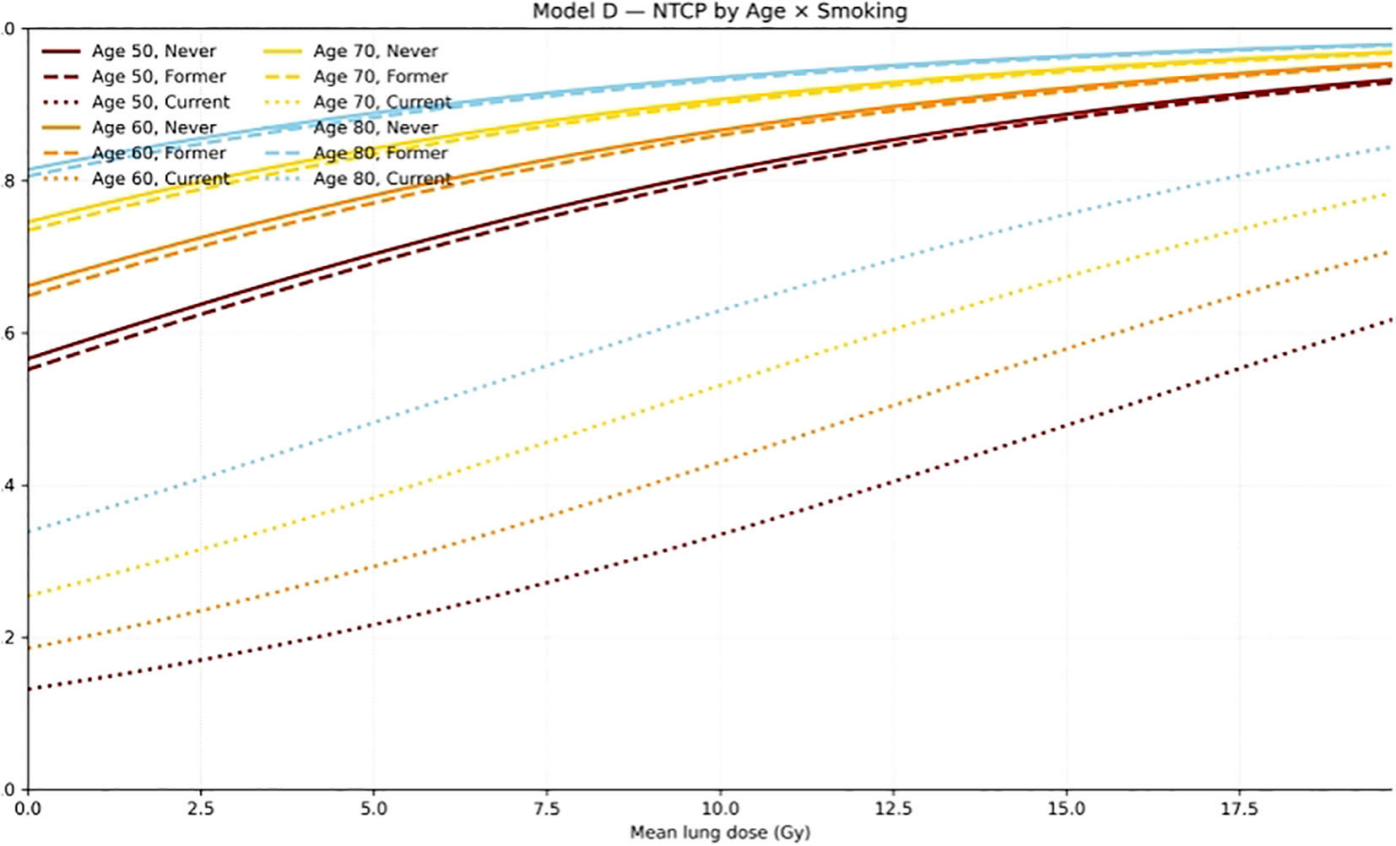
NTCP dose–response curves of the final simplified local model (model D) stratified by age and smoking status. The final APPELT model (model D) demonstrates that the probability of radiation pneumonitis (NTCP) increases with mean lung dose (MLD, Gy) across different age and smoking groups. Colors indicate age (50, 60, 70, and 80 years), while line types represent smoking status (solid, never smoker; dashed, former smoker; dotted, current smoker).

**Figure 4** illustrates the dose–response relationship of Model D stratified by age and smoking status. Predicted NTCP increased monotonically with increasing MLD and was consistently higher in older patients. Current smokers showed the lowest predicted risk across the MLD range, whereas former and never smokers had similar curves. In addition, the positive V30 coefficient indicated that greater medium-dose lung exposure was associated with higher RP risk.

### 3.4 External validation of the final simplified local model

#### 3.4.1 Discrimination and overall performance

**Figure 5:**
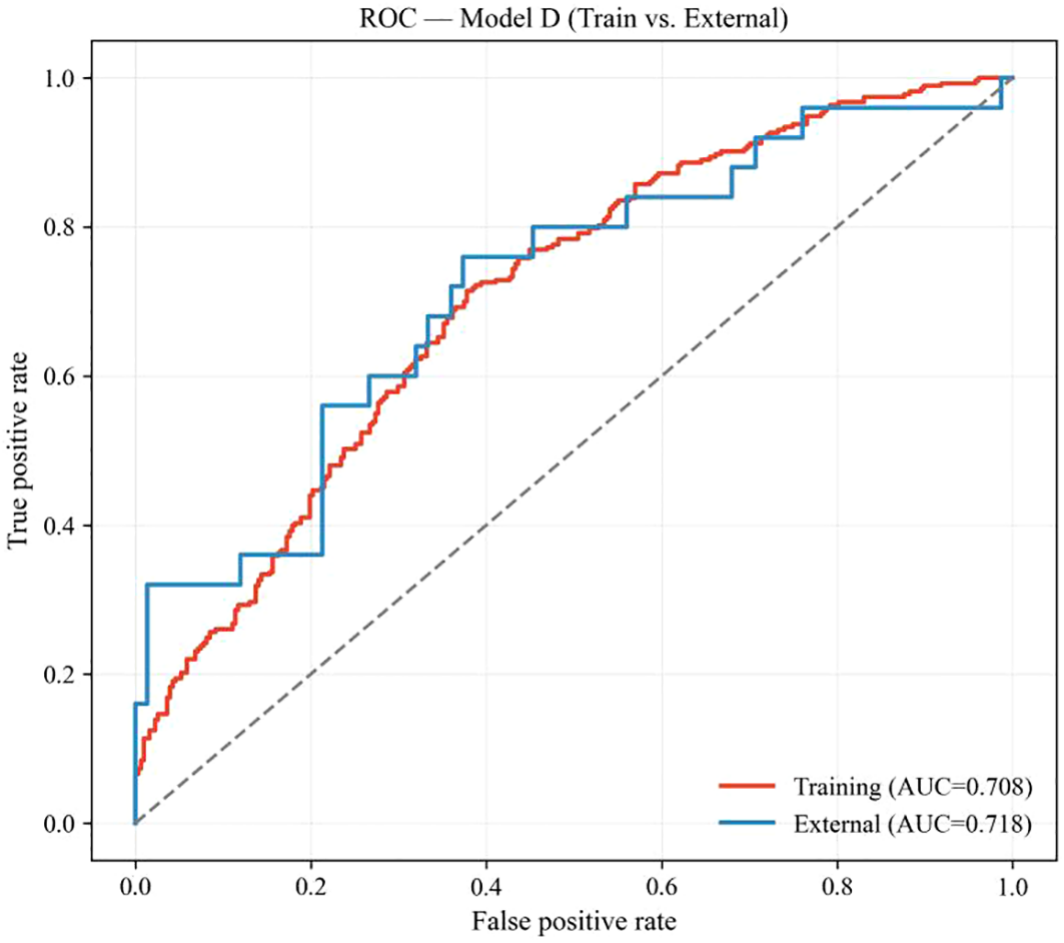
Receiver operating characteristic (ROC) curves of the final simplified local model (model D) in the development and external validation cohorts. Discrimination of Model D was maintained in the external cohort, with similar AUC values in the development and validation datasets.

The final simplified local model (Model D), with all coefficients fixed from the development cohort, was directly applied to the external validation cohort (n = 100; event rate, 25.0%). Discrimination was preserved, with an AUC of 0.708 in the development cohort and 0.718 in the external cohort (bootstrap 95% CI, 0.576–0.831) (**Figure 5**). Overall prediction error was also similar between cohorts, with Brier scores of 0.215 and 0.207, respectively. Detailed performance metrics for Model D in both cohorts are provided in **Supplementary Table 3**.

#### 3.4.2 Calibration in the external cohort

**Figure 6:**
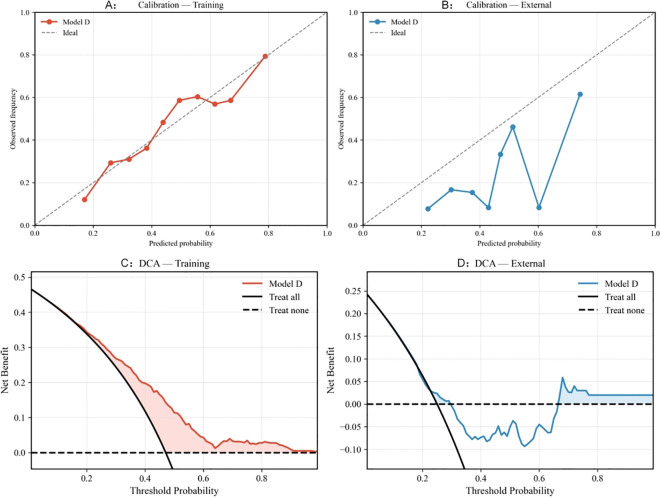
Calibration and decision curve analysis of the final simplified local model (Model D) in the development and external validation cohorts. **(A)** Calibration plot in the development cohort. **(B)** Calibration plot in the external validation cohort. **(C)** Decision curve analysis in the development cohort. **(D)** Decision curve analysis in the external validation cohort. Net benefit represents the clinical value of using the model to guide intensified RP risk-adapted management across threshold probabilities.In the decision curve plots, the solid black curve represents the “treat-all” strategy, whereas the thick black dashed horizontal line represents the “treat-none” strategy.

In the development cohort, Model D showed near-ideal apparent calibration, with a calibration-in-the-large (CITL) close to 0 and a calibration slope close to 1. In the external cohort, the calibration slope remained close to 1 (1.133), whereas the intercept shifted downward (CITL = −1.043), together with a Hosmer–Lemeshow χ² of 28.76 (P = 3.5 × 10^-4^). These findings indicate that the model preserved ranking ability but overestimated absolute RP risk in the lower-incidence external population. This pattern is consistent with a baseline-risk difference between cohorts and suggests that intercept recalibration may be sufficient when transferring the model to settings with lower event rates. In the present external validation, however, no recalibration was performed, because the aim was to evaluate the transportability of the locked model with all coefficients fixed from the development cohort. For calibration visualization in the external cohort (**Figure 6B**), four groups were used because of the limited sample size and event count.

#### 3.4.3 Decision curve analysis

Decision curve analysis showed sustained net clinical benefit across commonly used threshold probabilities in the development cohort (**Figure 6C**). In the external cohort, the magnitude of net benefit was smaller, but positive net benefit was observed mainly within the low-to-intermediate threshold range (approximately 0.05–0.29) (**Figure 6D**). Specifically, the net benefit of Model D in the external cohort was 0.168 at a threshold probability of 0.10, 0.119 at 0.15, and 0.023 at 0.25, approaching zero at around 0.30. Given the limited sample size and event count in the external cohort, these decision-curve findings should be interpreted cautiously.

## 4 Discussion

In the context of contemporary intensity-modulated radiotherapy (IMRT), we externally evaluated and updated two representative radiation pneumonitis (RP) prediction models, QUANTEC and APPELT, and further developed a final simplified local model (Model D). In our cohorts, the incidence of grade ≥2 RP was 30.6% in the development cohort and 25.0% in the external cohort. The original QUANTEC model showed modest discrimination but substantial underestimation of absolute RP risk, indicating marked miscalibration in our population. Recalibration of the intercept and MLD coefficient corrected this systematic calibration error, as expected, without altering discrimination (AUC 0.666), because recalibration primarily adjusts absolute risk estimates rather than patient ranking. By contrast, the original APPELT model showed both poor calibration and limited discrimination (AUC 0.628) when directly applied. After closed testing and stepwise simplification, the final simplified local model (Model D), incorporating age, stage, smoking status, tumor location, pulmonary comorbidity, NLR, SII, V30, and MLD, showed good apparent performance in the development cohort (AUC 0.708, Brier 0.215, calibration intercept ≈0, calibration slope ≈1, Hosmer–Lemeshow P = 0.599). In the external cohort, Model D showed similar discrimination and overall prediction error to those observed in the development cohort (AUC 0.718, bootstrap 95% CI 0.576–0.831; Brier 0.207), suggesting preserved ranking ability. However, calibration analysis showed a downward intercept shift (CITL −1.043) with a calibration slope close to 1 (1.133), together with a significant Hosmer–Lemeshow test (P = 3.5 × 10^-4^), indicating overestimation of absolute RP risk in the lower-incidence external population rather than loss of discrimination. Given the limited sample size and event count of the external cohort, these findings should be interpreted as preliminary rather than confirmatory.

From both biological and methodological perspectives, medium-dose lung burden, particularly V30, emerged as a relatively stable predictor of symptomatic RP in the IMRT era. Multiple studies have shown that V30/V40/V50 and MLD are consistently associated with grade ≥2 RP, whereas the predictive value of V5 is less robust and often depends on treatment technique and lung-volume definition. Bilateral or ipsilateral lung V30 has been identified as an independent predictor of RP, and real-world studies involving concurrent chemoradiotherapy and subsequent systemic therapy have also shown persistent associations for V30–V50 and MLD. Comparative analyses further suggest that medium-dose metrics may outperform “low-dose bath” parameters in IMRT populations, while earlier 3D-CRT data similarly supported the value of ipsilateral V20–V30 for risk stratification (14–20). These observations are consistent with our final model, in which V30 and MLD were jointly retained, supporting the biological plausibility and statistical robustness of medium-dose constraints, whereas V5 may be more susceptible to confounding by planning technique and volume definition.

Our findings were also broadly consistent with previous external validation studies. A likely explanation for the marked discrepancy between the observed RP risk in our cohort and the predictions from the original QUANTEC and APPELT models is the substantial heterogeneity between historical model-development cohorts and contemporary IMRT populations. The original models were developed in an earlier treatment era dominated by three-dimensional conformal radiotherapy, whereas current thoracic radiotherapy practice is largely based on IMRT and is increasingly delivered in combination with multimodal treatment. Such shifts may alter lung dose distributions, baseline event incidence, and the effects of individual predictors. In addition, differences in case mix, supportive care, toxicity ascertainment, and regional treatment patterns may further limit direct model transportability. Therefore, the marked underestimation observed in our cohort likely reflects baseline-risk shift and predictor-effect heterogeneity rather than simple failure of the historical models themselves. Consistent with this interpretation, the QUANTEC model required recalibration to improve calibration in the local population, whereas the APPELT framework required a more extensive updating pathway, resulting in recalibrated and simplified Appelt-derived models (Models A–C) before development of the final simplified local model (Model D) (9).

Importantly, Model D integrated immune-inflammatory markers (NLR and SII) with DVH parameters (V30 and MLD), thereby capturing the combined contribution of inflammatory susceptibility and medium-dose lung burden in the era of radiotherapy plus immunotherapy. Approximately 50.6% of patients in our cohort received PD-1/PD-L1 inhibitors, whereas the original QUANTEC and APPELT models were developed before these agents became part of routine lung cancer care (21). This shift in treatment exposure may partly explain the mismatch between historical model coefficients and the absolute RP risk observed in our population. Such an interpretation is biologically plausible, as prior studies have reported increased pulmonary toxicity in patients receiving PD-1 blockade in the setting of prior thoracic radiotherapy. In parallel, several studies have supported associations between NLR/SII and thoracic radiotherapy- or immunotherapy-related lung toxicity, and these biomarkers are attractive because of their accessibility, cost-effectiveness, and potential generalizability (22). Notably, our model also suggested a potentially lower RP risk among current smokers. Similar signals have been reported in some Chinese cohorts (22–25), whereas opposite findings have been observed in Western populations (26–28). These discrepancies may reflect differences in regional biology, treatment patterns, or residual confounding, and they should be interpreted cautiously pending validation in larger prospective cohorts.

An important methodological implication is that the improved calibration observed after updating the QUANTEC model and Appelt Models A/B should not be overinterpreted as evidence of stronger overall model performance. For recalibration procedures, improved calibration is expected by design because the intercept and/or slope are re-estimated to better match the target population, whereas discrimination is not expected to materially improve. The main value of these updating steps therefore lies in correcting systematic miscalibration and improving agreement between predicted and observed absolute risk. By contrast, the modest gain in discrimination observed with Model D likely reflects the incorporation of additional clinically relevant predictors beyond the original frameworks.

Although discrimination appeared broadly preserved across centers, absolute probability calibration was less stable in the lower-incidence external population. The negative CITL, together with a calibration slope close to 1, suggests that the main transportability issue was a shift in baseline risk rather than substantial distortion of predictor effects. From a practical perspective, this pattern implies that simple recalibration, particularly intercept adjustment, may be sufficient to realign predicted probabilities to local event rates without materially affecting discrimination. Accordingly, the model may be used to preserve relative risk ranking across centers, but local recalibration should be considered before applying it to absolute risk-based clinical decision-making in a new population. This distinction is important because preservation of ranking ability does not guarantee accurate absolute risk estimation when models are transferred to new clinical settings.

This study has several limitations. First, model development was conducted in a single center and external validation was performed in only one independent center, which may introduce selection bias and limit generalizability. Second, the external validation cohort was relatively small (n = 100, with 25 RP events). According to the methodological framework proposed by Riley et al. for prediction model validation, larger numbers of outcome events are generally desirable to obtain more precise estimates of model performance, particularly calibration. Therefore, the external validation results should be interpreted cautiously and regarded as preliminary rather than definitive evidence of transportability, and they are insufficient to support firm conclusions regarding broader generalizability. In addition, several potentially relevant predictors, such as detailed pulmonary function parameters and additional immune-related biomarkers, were unavailable for inclusion. Finally, the modest explained variance (AUC ≈0.71–0.72, Nagelkerke R² ≈0.19–0.20) indicates that residual unexplained risk remains. Future studies should validate the model in larger multicenter cohorts and explore integration of radiomics, multi-omics, and dynamic inflammatory biomarkers to further improve predictive performance.

### 4.1 Clinical implications

The final model enables individualized RP risk estimation before treatment planning. High-risk patients may benefit from tighter V30/MLD constraints, optimized field arrangements, and closer follow-up, whereas low-risk patients may be candidates for more intensive tumor-directed treatment while maintaining acceptable toxicity risk. After local recalibration and validation in larger independent cohorts, the model may serve as a candidate framework for individualized risk communication and treatment-planning trade-offs.

## Conclusion

We performed external evaluation and updating of the QUANTEC and APPELT models in a contemporary Chinese IMRT cohort and further developed a final simplified local model (Model D) incorporating age, stage, smoking status, tumor location, pulmonary comorbidity, NLR, SII, V30, and MLD. Updating of the existing models improved calibration as expected, without material change in discrimination. Compared with the traditional models, Model D showed better apparent overall performance in the development cohort. Because the external validation cohort was limited in size, these findings should be regarded as preliminary and should not be overinterpreted. Further large-scale multicenter validation is needed before broader clinical application.

## Data Availability

The original contributions presented in the study are included in the article/**Supplementary Material**. Further inquiries can be directed to the corresponding authors.
