# Supplementary material for: External validation and updating of NTCP models for radiation pneumonitis: QUANTEC, Appelt, and a local simplified model
Source: Front Oncol. 2026 Apr 28;16:1777999. doi: 10.3389/fonc.2026.1777999 (PMC13160778; doi:10.3389/fonc.2026.1777999)
Supplement: Supplementary file 1 [file DataSheet1.docx]

**Supplementary**

**Table S1 Comparison of CTCAE v5.0 Radiation Pneumonitis Grading and RGS (Radiation-induced Lung injury Grading Scale) Density-Based Criteria**

| Grade | CTCAE v5.0 (Radiation Pneumonitis) | RGS (Radiation-Induced Lung Injury) |
| --- | --- | --- |
| 0 | No pneumonitis detected. | RGS 0: No radiologic changes on CT. |
| 1 | Asymptomatic; clinical or diagnostic observations only; intervention not indicated. | RGS 1 (< 4 segments): Ground-glass opacities; mild consolidation (nodular, patchy, confluent); ipsilateral pleural effusion < 1 cm; volume loss; pleural thickening; interstitial changes or fibrosis. |
| 2 | Symptomatic; medical intervention indicated; limiting instrumental ADL. | RGS 2 (4–5 segments): Ipsilateral pleural effusion 1–2 cm; ground-glass opacity; moderate consolidation; volume loss; pleural thickening; interstitial changes or fibrosis. |
| 3 | Severe symptoms; limiting self-care ADL; oxygen indicated. | RGS 3 (> 5 segments): Ipsilateral pleural effusion > 2 cm; ground-glass opacity; severe consolidation; volume loss; pleural thickening; interstitial changes or fibrosis. |
| 4 | Life-threatening respiratory compromise; urgent intervention indicated (e.g., intubation). | — |
| 5 | Death. | — |

**Figures S1.**

Representative axial CT images with overlaid RGS density maps for Radiation‐Induced Lung Injury (RILI) Grades 1–5 (CTCAE v5.0), illustrating the progression from mild to severe injury:Grade 1: Minimal, focal ground‐glass opacity near the radiation field.Grade 2: Moderate ground‐glass opacity extending beyond the lesion core.Grade 3: Dense consolidation with early fibrotic strands.Grade 4: Extensive consolidation and marked fibrotic changes.Grade 5: Diffuse, bilateral lung involvement with severe fibrosis and volume loss.


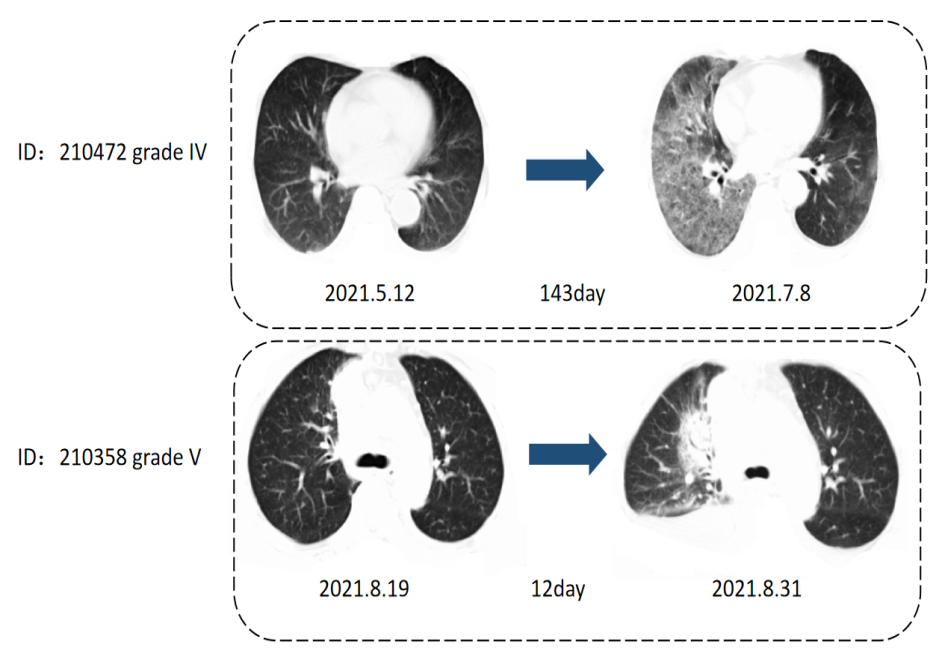

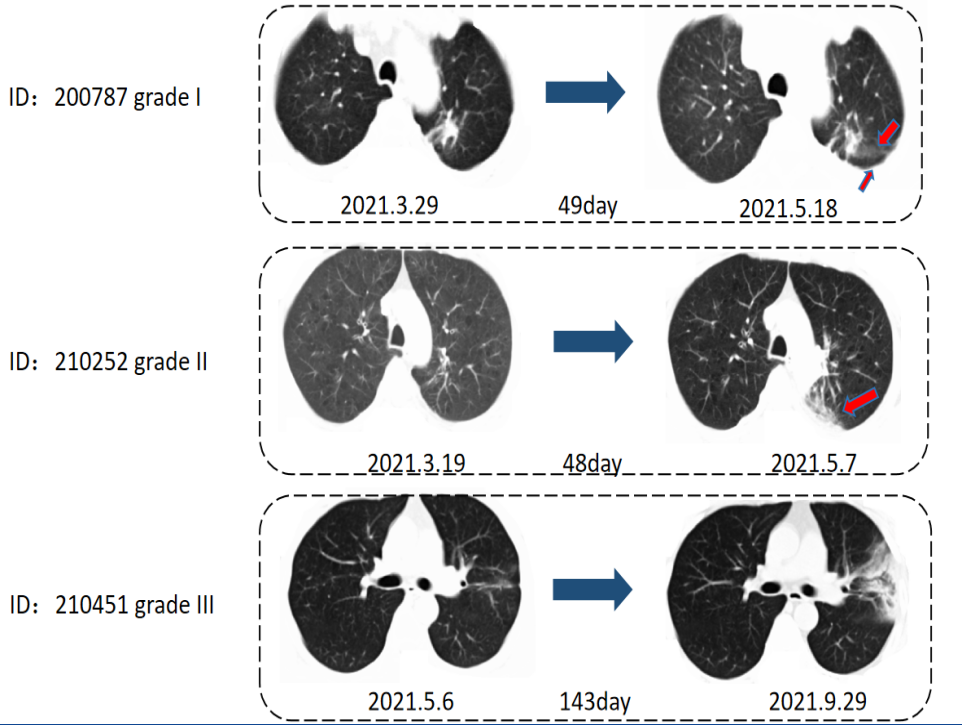


**Table S2.** Detailed treatment regimen distributions

| Variable | Total (n=680) | Training cohort (n=580) | External validation (n=100) | P value |
| --- | --- | --- | --- | --- |
| Chemotherapy |  |  |  | 0.08 |
| No | 186 (27.35%) | 151 (26.03%) | 35 (35.00%) |  |
| Yes | 494 (72.65%) | 429 (73.97%) | 65 (65.00%) |  |
| Induction chemotherapy |  |  |  | <0.05 |
| No | 533 (78.38%) | 484 (83.45%) | 49 (49.00%) |  |
| Yes | 147 (21.62%) | 96 (16.55%) | 51 (51.00%) |  |
| Concurrent chemotherapy |  |  |  | <0.05 |
| No | 437 (64.26%) | 351 (60.52%) | 86 (86.00%) |  |
| Yes | 243 (35.74%) | 229 (39.48%) | 14 (14.00%) |  |
| Adjuvant chemotherapy |  |  |  | <0.05 |
| No | 584 (85.88%) | 516 (88.97%) | 68 (68.00%) |  |
| Yes | 96 (14.12%) | 64 (11.03%) | 32 (32.00%) |  |
| Immunotherapy |  |  |  | 0.58 |
| No | 320 (47.06%) | 276 (47.59%) | 44 (44.00%) |  |
| Yes | 360 (52.94%) | 304 (52.41%) | 56 (56.00%) |  |
| Induction immunotherapy |  |  |  | <0.05 |
| No | 570 (83.82%) | 510 (87.93%) | 60 (60.00%) |  |
| Yes | 110 (16.18%) | 70 (12.07%) | 40 (40.00%) |  |
| Concurrent immunotherapy |  |  |  | 0.63 |
| No | 630 (92.65%) | 539 (92.93%) | 91 (91.00%) |  |
| Yes | 50 (7.35%) | 41 (7.07%) | 9 (9.00%) |  |
| Adjuvant immunotherapy |  |  |  | 0.07 |
| No | 372 (54.71%) | 326 (56.21%) | 46 (46.00%) |  |
| Yes | 308 (45.29%) | 254 (43.79%) | 54 (54.00%) |  |

**Table S3.** Performance of the final localized APPELT model (Model D) in the training and external validation cohorts

| Cohort | n | Events, n (%) | AUC | Brier score | Calibration intercept | Calibration slope | Hosmer–Lemeshow χ² | p-value |
| --- | --- | --- | --- | --- | --- | --- | --- | --- |
| Training | 580 | 178 (30.7%) | 0.708 | 0.215 | 0.003 | 1.001 | 6.81 | 0.557 |
| External | 100 | 25 (25.0%) | 0.718 | 0.207 | -1.043 | 1.133 | 28.76 | <0.001 |

note：AUC, area under the receiver operating characteristic curve; Brier, Brier score for overall prediction error; Calibration intercept (CITL), ideal value = 0 (positive values indicate systematic underestimation of risk); Calibration slope, ideal value = 1 (slopes <1 indicate overfitting, >1 indicate underfitting); Hosmer–Lemeshow (HL) χ² and p-value assess overall calibration (p>0.05 indicates acceptable fit).

**Table S4. Extended performance metrics for QUANTEC and APPELT models (local cohort)**

(A) QUANTEC model (Original, Recalibrated, Final)

| **Metric** | **Original** | **Recalibrated** | **Final** |
| --- | --- | --- | --- |
| Intercept | –3.87 | –1.742 | –1.652 |
| MLD coefficient | 0.126 | 0.164 | 0.155 |
| AUC | 0.666 | 0.666 | 0.666 |
| Hosmer–Lemeshow χ² | 1379.619 | 3.733 | 3.725 |
| p-value | <0.001 | 0.88 | 0.881 |
| Calibration intercept (CITL) | 3.287 | 0 | 0.007 |
| Calibration slope | 1.299 | 1 | 1.058 |

(B) APPELT model (stepwise updating from Original to Model D)

| **Metric** | **Original** | **Model A (Recalibrated)** | **Model B (Updated)** | **Model C (Simplified)** | **Model D (Final)** |
| --- | --- | --- | --- | --- | --- |
| Intercept | –4.76 | –1.7575 | –1.127 | –1.7422 | –0.8714 |
| MLD coefficient | 0.138 | 0.138 | 0.085 | 0.1637 | 0.1199 |
| Smoke_former | –0.37 | –0.37 | –0.2279 | – | –0.0572 |
| Smoke_current | –0.48 | –0.48 | –0.2956 | – | –2.15 |
| Comorbidity | 0.82 | 0.82 | 0.5051 | – | –0.0626 |
| Age | 0.51 | 0.51 | 0.3141 | – | 0.0405 |
| SeqChemo | 0.47 | 0.47 | 0.2895 | – | – |
| Loc_midinf | 0.63 | 0.63 | 0.388 | – | – |
| Stage | – | – | – | – | –0.2493 |
| NLR | – | – | – | – | –0.1669 |
| SII | – | – | – | – | 0.0006 |
| V30 | – | – | – | – | 0.0498 |
| AUC | 0.6275 | 0.6275 | 0.6275 | 0.6659 | 0.7075 |
| Hosmer–Lemeshow χ² | 2614.248 | 18.795 | 8.422 | 3.934 | 6.428 |
| p-value | <0.001 | 0.016 | 0.393 | 0.863 | 0.599 |
| Calibration intercept | 1.805 | –0.045 | 0 | 0 | 0 |
| Calibration slope | 0.616 | 0.616 | 1 | 1 | 1 |
